# ‘We Did the Very Best We Could for the Residents’: A Thematic Analysis of Work Experience in Swedish Nursing Homes During the COVID‐19 Pandemic

**DOI:** 10.1111/scs.70052

**Published:** 2025-07-04

**Authors:** Viktor Carlsson, Ingrid L. Gustafsson, Harald Berg Ljungdahl, Anna Sofia Bratt

**Affiliations:** ^1^ Faculty of Health and Life Sciences, Department of Psychology Linnaeus University Växjö Sweden; ^2^ Regional Department of Competence in Family Medicine and Primary Health Care Region Kronoberg Växjö Sweden; ^3^ Faculty of Caring Science, Work Life and Social Welfare University of Borås Borås Sweden; ^4^ Faculty of Health and Life Sciences, Research Secretary at Centre of Interprofessional Collaboration within Emergency Care (CICE) Linnaeus University Växjö Sweden

**Keywords:** COVID‐19, experience, nursing, nursing homes, older adult, qualitative, thematic analysis

## Abstract

**Aim:**

The aim of this study was to deepen our understanding of how Swedish nursing staff experienced work in nursing homes (NHs) for older adults during the COVID‐19 pandemic.

**Methods:**

An inductive qualitative design with an experiential focus was employed. Data were collected through interviews and analysed using reflexive thematic analysis. The participants were employed at five NHs located in four municipalities and comprised two registered nurses, eight licensed practical nurses, and one licensed practical nurse trainee.

**Ethical Issues and Approval:**

This study was approved by the Swedish Ethical Review Authority. Participation was voluntary, and all participants provided their written informed consent.

**Results:**

The analysis resulted in two themes: *A crucible of pressure and uncertainty* and *Being there for one another*. The first theme described an intertwined experience of pressure and uncertainty, with heightened workloads, increased responsibility, frequent changes and elevated risk. Leadership and organisational factors played significant roles and could either exacerbate or alleviate experiences of pressure and uncertainty. The second theme described the willingness and effort of nursing staff to support one another and the residents. It included recognising one another's situations, showing empathy, and providing help. Experiences of distress were evident in both themes and included stress, worry and guilt.

**Conclusions:**

These findings add to existing knowledge of the experience of nursing staff in NHs in Sweden during the COVID‐19 pandemic. They highlight a distressing situation as well as a sense of community. Additionally, the findings emphasise the importance of supportive leadership and clear directives, which can inform future policy and practice in similar situations.

## 1 Introduction

Nursing homes (NHs) for older adults came into focus during the COVID‐19 pandemic (hereafter, ‘the pandemic’), revealing a lack of preparedness to protect their residents [1, 2, 3]. During the first wave of the pandemic in Sweden, the spread of COVID‐19 to elderly care services was higher than in other Nordic countries [4], and compared to older adults with home care or living independently, NH residents had the highest excess mortality [5]. NHs in Sweden faced criticism for taking insufficient action to safeguard their residents [3], and their staff felt accused by the mass media and residents' relatives for their handling of COVID‐19 [6]. The attention directed towards NHs also highlighted their working conditions, underscoring the low status of the work [2, 3].

At the onset of the pandemic, NHs frequently needed to adjust their routines and decisions [7], and their staff experienced a lack of relevant skill and knowledge [1]. Research conducted both in Sweden [6, 8] and internationally [1, 9] highlights the challenges and negative consequences experienced by staff and residents during the pandemic. For instance, residents were prohibited from receiving visits from loved ones and interaction with other residents was limited, leading to feelings of loneliness and sadness [8].

Furthermore, workforce shortages in NHs were already critical before the pandemic, and when personnel fell ill or exhibited cold symptoms, they had to quarantine at home, burdening the remaining personnel [6]. Protective equipment was limited at the beginning of the pandemic and the staff felt abandoned [1, 2, 6]. However, they supported each other, developed new skills, and took on new responsibilities and roles [6, 10].

The experiences of Swedish NH staff during the pandemic have been explored in qualitative research [6, 11]. However, the number of studies is limited, and understanding these experiences is crucial for better preparedness for similar future situations. Also, given the criticism directed towards Swedish NHs [3] and the scrutiny and high demands faced by their staff [6], further investigation of the experiences of Swedish nursing staff in NHs during the pandemic is warranted. The aim of this study was accordingly to deepen our understanding of how Swedish nursing staff experienced work in NHs for older adults during the COVID‐19 pandemic. The specific research question was: How have Swedish nursing staff experienced work in NHs for older adults during the COVID‐19 pandemic?

## 2 Methodology

In the present study, a qualitative design with an experiential focus [12, 13] was employed. The research was grounded in a critical realist position, acknowledging participants' experiences as their interpretations of reality, situated within their individual contexts [13]. The approach of this study was inductive, and the analysis was not guided by a specific theoretical framework. However, researchers always bring their unique perspectives to the analysis and interpret the data through the lens of their own experiences and contexts. The chosen method for analysis was reflexive thematic analysis, as outlined by Braun and Clarke [13], which emphasises this subjectivity of the researchers. Reflexivity, including reflection on how the researchers' values and practices affect the research, is therefore central to this method. This article complies with the Standards for Reporting Qualitative Research (SRQR) [14].

### 2.1 Participant Recruitment

The inclusion criteria were as follows: (1) Nursing staff currently employed at an NH. This included registered nurses as well as nursing staff who worked closest to the residents, attending to their daily care. (2) Worked at the NH for at least 2 months during the period from March 2020 to March 2021. (3) Speak understandable Swedish without needing an interpreter.

In southern Sweden, we contacted operations managers of NHs or higher‐level managers responsible for NHs in a municipality. Contact was established via e‐mail or telephone. Managers who expressed interest in having their NHs participate received additional information and were encouraged to share details of the study with their personnel. Interested personnel could then contact one of the researchers (VC or HBL) for further information and to sign up for the study. Because the invitations were extended by the managers, our research group lacks specific data on the total number of individual NHs or presumptive participants contacted. Also, as this meant that the authors did not choose which specific individuals to invite to the study, the sample may be considered a convenience sample [15]. However, the researchers did not predefine any attributes to consider when recruiting participants except for the inclusion criteria.

### 2.2 Participants

Fourteen participants were initially recruited for the study and signed informed consent. However, one participant did not respond to the interview scheduling request, and two were excluded because they were not considered nursing staff. Of the excluded participants, one held a managerial position at an NH, while the other had work assignments that did not directly involve nursing. Consequently, 11 nursing staff participants (10 females and one male) participated in the study: two were registered nurses, eight were licensed practical nurses (English title according to the Swedish Medical Subject Headings [MeSH] [16]), and one was in training to become a licensed practical nurse. The licensed practical nurses, including the one in training, worked closest to the residents in their daily care. All participants had been working at their respective NHs since the start of the pandemic, and their total NH work experience ranged from three to 37 years. They worked in five NHs situated in four municipalities. Four of the NHs were municipally run and one was operated by a private company. Of the participants, five worked at a single NH, two NHs had two participants each, and the remaining two NHs had one participant each.

### 2.3 Data Collection

Data were collected through interviews, a well‐suited method when focusing on participants' experiences [15]. The interviews were conducted from October 2021 to April 2022 and were based on two broad open questions: ‘Could you share your experiences about how work has been at [name of NH] during the COVID‐19 pandemic?’ and ‘Could you describe what it has been like for you to work at [name of NH] during the COVID‐19 pandemic?’. Follow‐up questions were employed to deepen our understanding of the participants' experiences, and relevant areas that the participants touched upon could be further explored. Additionally, background data concerning profession and work experience were gathered. Six of the included interviews were conducted by HBL and five by VC. Four interviews were conducted by telephone and seven were conducted in person at the NHs where the interviewees worked. The interviews lasted 16–46 min and were audio recorded; the recordings were transcribed verbatim. HBL transcribed the interviews he conducted, while a secretary transcribed the interviews conducted by VC.

### 2.4 Analysis

The data were analysed using reflexive thematic analysis, following the approach outlined by Braun and Clarke [13, 17]. The analysis was inductive, had an experiential focus [12, 13], and unfolded in six phases [13, 17]. In the first phase, familiarisation, all transcripts were read by VC, ILG, and ASB, while HBL reviewed the transcripts of the interviews that he conducted. In the second phase, all transcripts were coded for meaning units by VC. In the coding, the focus was on meaning that was expressed at a manifest level (semantic meaning), capturing the participants' expressions of their individual experiences. In the third phase, the codes were clustered into preliminary themes, and in phase four, these were developed, reviewed and cross‐checked against the data. This included new readings of the interviews in their entirety. In phase five, the themes were further refined and named. Throughout phases three to five, which were mainly conducted by VC, the analysis and issues of reflexivity, including our preunderstanding, backgrounds and roles as researchers, were regularly reflected upon and discussed between VC, ILG and ASB. In this collaborative process, the preliminary themes were reviewed and adjusted several times. Efforts were made to create cohesive themes, capturing a central aspect of the participants' experiences. All authors agreed on the final themes. Finally, in phase six, the report was produced. Quotations included in the report are slightly adjusted for readability. If text is removed from the quotation, this is marked with […].

VC and ASB are licensed psychologists, while ILG is a registered nurse anaesthetist; all three are researchers with experience in qualitative methodology. HBL is a licensed psychologist but was a master's student in psychology at the time of data collection. This means that three of the four authors were psychologists. Although they had immersed themselves in literature, had some relevant personal connections and experiences in the field, and HBL had prior experience working part‐time in elderly care services, they came to the area of nursing with somewhat of an outside perspective. The nursing perspective in this study was supported by ILG, who provided more of an inside perspective. As mentioned earlier, the analysis was inductive, and there was no specific theoretical framework from nursing or psychology that guided the analysis. However, the point of view of the psychologists when approaching this work can broadly be described as stemming from a humanistic stance, with a focus on human experience and a holistic understanding of these phenomena [18].

### 2.5 Ethical Considerations

This study was approved by the Swedish Ethical Review Authority (No. 2021–03698). The research adhered to the declaration of Helsinki [19]. Participation was voluntary, and all participants provided their written informed consent. All collected material was stored securely, and confidentiality was ensured. Identifying features were removed from the transcripts, which were assigned a number. Only VC and HBL, who conducted the interviews, had access to the names of the participants.

## 3 Results

Our analysis resulted in two themes: *A crucible of pressure and uncertainty* and *Being there for one another*. Most participants had only had a few residents — in some cases, none — infected with COVID‐19 at their NHs, although some had experience with a larger number of infected residents.

### 3.1 A Crucible of Pressure and Uncertainty

This theme describes an intertwined experience of pressure and uncertainty. The experience generally entailed increased workload and responsibility in a changing and uncertain high‐stakes situation, and several participants described associated feelings of distress. The experience of pressure and uncertainty was most evident at the beginning of the pandemic and gradually diminished as the situation normalised and stabilised. However, for some participants, aspects of the experience persisted. The following quotation exemplifies this intertwined experience:

Well, it has often been very unsettling, and it has been quite tough, especially when there are more [residents] who get colds and all that. And then you have to run and check on them all the time, and you've been listening to the TV all the time, and you hear that it just keeps rising and rising and rising. So, it has been very tough not really knowing how it will turn out. Everything is so very new for us, so you feel very mentally tired when you come home, and, yes, pounding headaches, quite often to be more precise. (Participant 11)

This quotation illustrates the experience of facing increasing challenges on multiple fronts. Changes in routines occurred swiftly, and alongside the general sense of uncertainty brought about by the pandemic, there were in some cases uncertainty regarding what actions to take and who had the authority to decide on routines and protective measures. One participant gave the following description:

At the beginning there was a lot of unrest, a bit of panic, nobody quite knew how to handle it, and we didn't get any clear directives either. […] The manager just said ‘It's the [registered] nurses who make the decisions’. The [registered] nurses [said], ‘No, it's the care staff’, meaning it's us who decide if we need, say, protective gear or what needs to be done. It was all just chaotic. So, you feel, felt, a little panicked about it. (Participant 1)

In the participants' stories, it was obvious that leadership and organisational factors were important, and could either exacerbate or alleviate experiences of pressure and uncertainty. As indicated by the above quotation, feeling unheard by the manager or seeing that proper routines were not being implemented could be major sources of distress. However, most interviewees described positive leadership experiences at their NHs. It was experienced as supportive and helpful that managers genuinely listened, cared, and strived to meet individual needs. The managers or other organisational structures were often helpful in sorting the flow of information. The following quotation exemplifies the emotional impact of clear routines and trust in leadership. It describes a situation in which staff found out that they had their first COVID‐19‐infected resident, and the manager had experience of infected residents from another NH:

He [i.e., the manager] came down and explained how we should proceed. I felt quite safe about it. I thought he had experienced it once before, so I trusted him to know what he was doing. (Participant 4)

Substantial parts of the interviews concerned the use of protective equipment and following hygiene routines and restrictions. However, the extensive use of protective equipment had its drawbacks for staff. Face masks, in particular, posed challenges. Some interviewees reported feeling constricted and unable to breathe properly while wearing one. The use of protective equipment also made the work more demanding and tiring, although over time, they became more accustomed to it. The following quotation illustrates the extensive use of protective measures and the mental tension arising from striving to avoid mistakes, adding to the experience of pressure and uncertainty:

You also knew that the person you were caring for was infected, so then you had to think extra carefully. Okay, how should we proceed now? Should we disinfect all the rooms meticulously to prevent any viruses from spreading? We had to restrict—nothing could leave the room [were the infected resident lived] and enter the corridor, everything had to stay in there. You couldn't forget any steps, and when you managed to remove the contamination: ‘Did I just carry the laundry through the entire corridor? I forgot to put it in a bag…’ Yes, you were constantly on high alert. (Participant 5)

In some cases, the staff wanted to use more protective equipment than was recommended at the time by local authorities or to implement more restrictive routines than advised by their organisation. Working hard to keep their residents and themselves safe, staff were concerned about being blamed if they spread the virus to the NH. One participant who worked at an NH that had not had any residents infected by COVID‐19 expressed this:

And the anxiety when there was such high mortality, as people were talking about—no one wanted to be the one who brought the infection into the facility. And it still lingers, it's not something that has gone away, I can tell you that. […] The worry and fear that one might become some kind of scapegoat, even though no one thinks that way, but one tends to think so oneself. (Participant 3)

The above quotation illustrates vulnerability, the risk of being held accountable for mistakes. It further illustrates the pressure and uncertainty experienced. Worries included not only the fear of spreading the virus to residents and colleagues but also concern about catching the virus at work and transmitting it to one's own family. This concern was particularly profound for those who had relatives who were susceptible to serious illness.

Another aspect that made the working situation even more pressing was the high prevalence of staff sick leave due to the low threshold for symptoms. This resulted in a higher workload for the remaining personnel, and even when substitutes could be found, they might not be able to assume the full workload of the regular staff.

Many described the work during the pandemic as a strain, and some mentioned consequences such as tiredness, stress, or headache. There were also some indications that the pressure and uncertainty at times led to increased distrust. It was sometimes questioned whether colleagues who stayed at home often or in certain situations were genuinely sick. In other cases, staff were instructed by superiors not to be honest with residents about the COVID‐19 situation in the facility, which felt ethically wrong.

### 3.2 Being There for One Another

This theme describes staff willingness and striving to support one another. It includes recognising one another's situations, showing empathy, and providing help. In moments when one's ability to help or to alleviate suffering was limited, feelings of distress or guilt emerged.

Within the staff, a sense of community and mutual support appears to have been integral to dealing with the challenges brought by the pandemic. Participants described efforts to help one another and noted that the hard work that took its toll on them could also, at times, bring the staff members closer together. One participant described the situation as follows:

We have managed very well, with a lot of cooperation and camaraderie. Now you forgot this, and then you fixed that—like that. We have tried to help one another all the time and as much as possible. (Participant 9)

The desire to support one another could sometimes lead to feelings of guilt in situations in which one was unable to help one's colleagues, such as when staying at home with cold symptoms. However, it was not always easy to evaluate whether what one felt in the morning were symptoms of a beginning cold or just a passing sensation, and misjudgments could further contribute to feelings of guilt.

Another important aspect of this theme was how the staff empathised with the residents. The implemented protective measures, restrictions, and reduced activities had extensive consequences for the residents that the staff had to witness. They often experienced that their actions put further strain on the residents and that the time available to care for their social and emotional needs was limited. One participant expressed it as follows:

Everyone who lives here is entitled to personal time. We didn't manage to fit that in, though. Because then you can have time for a walk, or to sit and talk or do something else they find pleasant. But we had to cancel that, there simply wasn't time for. […] So, it didn't feel right. They would have needed it even more when sitting isolated and alone. Then they would have needed even more stimulation from other people—if you had the time to sit and talk—if nothing else, just to feel that they're not completely alone. (Participant 4)

However, there were also many efforts made to reduce these negative consequences. Several interviewees described sometimes making minor deviations from the routines to improve communication or soothe residents. They also tried to compensate for the loss of contact with loved ones, for example, by spending more time talking with the residents or finding ways of facilitating communication between the residents and their loved ones. One participant described the following experience:

Well, we sat with them more… so we made a real effort to get them out on this walk that many of the relatives normally did. Then we had to take over that part instead […] You had to take the time. […] But I think we all understood, anyhow, that it still had to be done [even if there was a lack of time]. (Participant 7)

Some interviewees observed an increase in depressive symptoms among the residents as a result of lack of activity, being unable to meet loved ones, or other limitations in social interaction. The use of protective equipment also had a direct impact, which could be negative for the residents:

When you enter fully equipped—I mean, some residents can become very, very scared. And handling that—well, it's tough, it's not fun to do. Some took it really… they found it very difficult. And then it was tough for oneself as well, knowing that this person would become very anxious, not knowing what's happening, ‘who are you?’ [the resident may ask]. (Participant 1)

As described in the above quotation, witnessing these consequences could be emotionally distressing for the staff. Residents with dementia were especially vulnerable and often became frightened when faced with the use of protective equipment. For them, uncomfortable testing procedures were perplexing, and they did not understand why they had to undergo them. The use of face masks and face shields could also hinder communication, which could be frustrating for the residents:

Some residents have been able to express it: ‘Take off that crap, I don't believe in it’, they might say, ‘Why do you look like aliens?’ (Participant 3)

## 4 Discussion

The aim of this study was to deepen our understanding of how Swedish nursing staff experienced work in NHs for older adults during the COVID‐19 pandemic. The analysis resulted in two themes: *A crucible of pressure and uncertainty* and *Being there for one another*. The first theme described an intertwined experience of pressure and uncertainty, entailing heightened workloads, increased responsibility, frequent change, and elevated risk. Leadership and organisational factors played significant roles, either exacerbating or alleviating these pressures and uncertainties. The second theme described the willingness and efforts of nursing staff to support one another and the residents. This involved recognising one another's needs, showing empathy, and providing help. Experiences of distress were evident in both themes and included stress, worry, and guilt.

Protecting health and life lies at the heart of caregiving [20], and the participants in our study worked hard to follow directives and uphold protective measures. However, they were also troubled by worries about spreading the disease, in line with previous Swedish and international research [1, 6]. Our findings are also aligned with prior research emphasising high workload and strain [1, 6].

Apart from protection from health risks, a central aspect of caregiving is the alleviation of suffering. Caring is rooted in love, kindness, and a caring relationship [20]. As protective measures were implemented in the NHs, nursing staff witnessed the negative consequences faced by residents. Motivated to meet residents' emotional and social needs, they did their best to provide support and care. However, they faced numerous limitations, and not being able to cater to these needs was distressing. These results are also in line with previous research indicating feelings of troubled conscience due to limited ability to support and spend time with NH residents [6]. Moral challenges are an inevitable part of caregiving work, and personnel grapple with achieving balance between their personal moral principles and their professional responsibilities [21]. These challenges can serve as a source of deeper meaning in our work, and lay the groundwork for enhancing ethical competence, provided that staff allocate time for reflection. Addressing moral distress—defined as knowing the right course of action but facing institutional constraints that hinder its implementation ([22], p. 6)—requires dedicated time for reflection to develop ethical competence. Ethical competence encompasses the ability to do the right thing for patients and draws on ethical awareness and sound moral judgement [23]. In the ‘Art of Caring’ model, the authors illustrate how ethical dilemmas encountered in clinical practice can be shared and discussed with colleagues, using self‐compassion and reflection, which in turn enhance ethical competence and potentially reduce moral distress in healthcare providers [24]. Compassion is a relational process that can be directed from oneself to another, from another to oneself, or from oneself to oneself, i.e., self‐compassion [25]. While healthcare providers often direct compassion to others, fostering self‐compassion and being open to receiving compassion from others are equally vital. The ‘Art of Caring’ model can be applied in NHs, enhancing staff members' ethical competence and self‐compassion abilities.

The present results add to existing knowledge of the experiences of Swedish nursing staff in NHs during the pandemic, as reported in previous research [6]. Mutual support among NH staff appears to have been a crucial aspect of dealing with the pandemic, as evident in both our study and earlier research [6]. Furthermore, in the present study, leadership played a significant role in managing the challenges of the pandemic, and there were many instances of feeling supported and receiving clear guidance from management. However, experiences of not being supported by management, as described in earlier research [6], were also present. Leadership, or lack thereof, thus seems to have been an important factor for nursing staff in dealing with the pandemic in Swedish NHs. Another Swedish study on experiences from municipal health and social care, including NH staff, also emphasised the beneficial effect of experiencing support from superiors [11]. In our study, it was common for staff to experience low rates of COVID‐19 infections in their respective NHs. It is possible that experiences regarding leadership and infection rates may influence each other and that, for example, the pressure on leadership may increase with higher infection rates. International research has also shown that the pandemic was a strain and morally distressing for NH managers [7, 26].

### 4.1 Methodological Considerations

Yardley [27] proposes four key criteria for evaluating qualitative research: sensitivity to context, commitment and rigour, coherence and transparency, and impact and importance. Sensitivity to context is essential throughout the research process. In this study, the researchers demonstrated contextual awareness through familiarity with literature on NHs for older adults and experiences. Interviews were conducted with nursing staff working in NHs, situating the study within a broader, yet nuanced, context. The participants´ perspectives reflected diverse experiences within this setting. The interviewers committed to having an emphatic and respectful approach and broad open‐ended questions were used to capture the perspectives of the participants. We felt that the participants were able to speak freely and openly and were engaged in the interviews. As described earlier, three of the authors had somewhat of an outside perspective. Also, both of the interviewers were male while almost all of the participants were female. These aspects probably influenced the participants to some degree, and it may be that they would have felt further security in sharing their thoughts and experiences if the interviewers were more knowledgeable and involved in their direct working conditions, and perhaps also if they were of the same gender.

Regarding commitment and rigour, the research question was intentionally broad, exploring nursing staff work experiences during the pandemic, which meant that we sought to include participants with various experiences. As a result, participants in the study came from five NHs situated in four municipalities and consisted of both licensed practical nurses and registered nurses. This indicates a breadth of experiences in line with the research question. The first wave of the pandemic struck Sweden in the spring of 2020, followed by subsequent waves. The interviews for this study were conducted from October 2021 to April 2022. Although the research question did not focus specifically on a particular wave or time period, the intense initial phases of the pandemic still seemed vivid to the participants and stood out in the interviews. While the passing of time might have affected participants' memories, it may also have enabled time for reflection. As previously discussed in relation to our results, most participants had dealt with no or relatively few NH residents infected with COVID‐19. We can only speculate about the reasons for this, but it is possible that presumptive participants with different experiences, or their managers, may have been more reluctant to participate. However, experiences of a higher number of infected residents were also captured in the data, so that perspective was not omitted. Finally, the composition of the research team enabled a variety of perspectives to be considered throughout the analytic process.

Coherence and transparency: We consider the study as coherent in its overall design. Although not rooted in a specific theoretical framework from nursing or psychology, the ontology, background, research focus, and methodology are well aligned. In order to increase the transparency of our analysis and deepen the understanding of the findings, a variety of quotations from the participants were presented. We also engaged in reflexive reflections and discussions regarding how our personal positions might influence the results. Since the interviewers and the main conductor of the analysis were psychologists, there might have been aspects specific to nursing that we failed to acknowledge. On the other hand, this may also have resulted in more explicit exploration of areas that may have been taken for granted if the interviewer were a nurse experienced in working at NHs.

Impact and importance: The broad scope of the research question and collected data bolsters the transferability to similar nursing contexts but diminishes the transferability when it comes to specific professions, such as the experiences of either registered nurses or licensed practical nurses. Still, the findings highlight the importance of leadership in nursing settings, particularly during times of crisis, and may serve as a valuable point of reflection for future practice and policy.

## 5 Conclusions

The present results add to existing knowledge of the experience of NH nursing staff in Sweden during the COVID‐19 pandemic. They highlight a distressing situation as well as a sense of community. The staff faced significant uncertainty and high workload and struggled to meet the needs of the residents. Additionally, the findings emphasise the importance of supportive leadership and clear directives, which can inform future policy and practice in similar situations.

## Author Contributions

V.C., I.L.G., and A.S.B. contributed to the conceptualization and design of the study. V.C. and H.B.L. were responsible for data collection and project administration. V.C. conducted the formal analysis with support from the other authors (see Section 2 for details). The initial draft was primarily written by V.C., with contributions from I.L.G. and A.S.B. All authors reviewed, edited, and approved the final manuscript.

## Conflicts of Interest

The authors declare no conflicts of interest.

## Data Availability

The research data for this publication are not publicly available due to ethical restrictions.
